# Effects of Drying Temperature and Solvents on In Vitro Diabetic Wound Healing Potential of *Moringa oleifera* Leaf Extracts

**DOI:** 10.3390/molecules28020710

**Published:** 2023-01-11

**Authors:** Saima Muzammil, Jorddy Neves Cruz, Rabia Mumtaz, Ijaz Rasul, Sumreen Hayat, Muhammad Asaf Khan, Arif Muhammad Khan, Muhammad Umar Ijaz, Rafael Rodrigues Lima, Muhammad Zubair

**Affiliations:** 1 Department of Microbiology, Government College University (GCU), Faisalabad 38000, Pakistan; 2 Laboratory of Functional and Structural Biology, Institute of Biological Sciences, Federal University of Pará, Belém 66075-110, PA, Brazil; 3 Department of Bioinformatics and Biotechnology, Government College University (GCU), Faisalabad 38000, Pakistan; 4 Institute of Plant Breeding and Biotechnology, MNS-University of Agriculture, Multan 59300, Pakistan; 5 Department of Biotechnology, University of Sargodha, Sargodha 40100, Pakistan; 6 Department of Zoology, Wildlife and Fisheries, University of Agriculture, Faisalabad 38000, Pakistan

**Keywords:** antidiabetic, antioxidant, wound healing, phenolic compounds

## Abstract

The delayed healing of wounds among people with diabetes is a severe problem worldwide. Hyperglycemia and increased levels of free radicals are the major inhibiting factors of wound healing in diabetic patients. Plant extracts are a rich source of polyphenols, allowing them to be an effective agent for wound healing. Drying temperature and extraction solvent highly affect the stability of polyphenols in plant materials. However, there is a need to optimize the extraction protocol to ensure the efficacy of the final product. For this purpose, the effects of drying temperature and solvents on the polyphenolic composition and diabetic wound healing activity of *Moringa oleifera* leaves were examined in the present research. Fresh leaves were oven dried at different temperatures (10 °C, 30 °C, 50 °C, and 100 °C) and extracted in three solvents (acetone, ethanol, and methanol) to obtain twelve extracts in total. The extracts were assessed for free radical scavenging and antihyperglycemic effects using DPPH (2,2-diphenylpicrylhydrazyl) and α- glucosidase inhibition assays. Alongside this, a scratch assay was performed to evaluate the cell migration activity of *M. oleifera* on the human retinal pigment epithelial cell line. The cytotoxicity of the plant extracts was assessed on human retinal pigment epithelial (RPE) and hepatocellular carcinoma (Huh-7) cell lines. Using high-performance liquid chromatography, phenolic compounds in extracts of *M. oleifera* were identified. We found that an ethanol-based extract prepared by drying the leaves at 10 °C contained the highest amounts of identified polyphenols. *Moringa oleifera* extracts showed remarkable antioxidant, antidiabetic, and cell migration properties. The best results were obtained with leaves dried at 10 °C and 30 °C. Decreased activities were observed with drying temperatures of 50 °C and above. Moreover, *M. oleifera* extracts exhibited no toxicity on RPE cells, and the same extracts were cytotoxic for Huh-7 cells. This study revealed that *M. oleifera* leaves extracts can enhance wound healing in diabetic conditions due to their antihyperglycemic, antioxidant, and cell migration effects. The leaves of this plant can be an excellent therapeutic option when extracted at optimum conditions.

## 1. Introduction

Diabetes is a global health concern, with an estimated 537 million individuals suffering from this disease worldwide, and the number is anticipated to reach 643 million by 2030 and 784 million by 2045. Over 81% of the people affected by diabetes are living in economically developing countries. Diabetes accounts for an estimated 966 billion dollars in global healthcare costs in 2021, representing a 316% rise during the last 15 years [1]. Diabetes mellitus leads to the progression of various micro and macrovascular problems and non-healing skin ulcers [2]. Wounds in diabetic patients are prone to infections, are slow to recover, and can last for months, making them a significant healthcare burden [3,4,5,6]. High blood glucose in diabetic patients causes increased infection development, prolonged inflammation, and reactive oxygen species production, associated with impaired proliferation and the remodeling stages and reduced strength of the wounded area [7]. 

Clinicians and wound care staff in developed countries are using advanced therapies such as hyperbaric oxygen therapy, negative pressure therapy, antibiotics, and growth factors to improve healing in diabetics [8]. Unfortunately, the available treatments are limited by their disadvantages, including high costs, allergic reactions, and microbial resistance. Moreover, these therapies are not available in developing countries, so to minimize the toxic effects, an effective and safe alternative from natural resources must be found [9].

Medicinal plants and herbal preparations represent a significant portion of the global healthcare market. In developing countries, 80% of people use traditional medicines due to their easy availability, low cost, and effectiveness. Various ailments can be cured with herbal remedies, including ulcers, skin infections, inflammation, and diabetic wounds [10]. Plants used to treat wounds provide disinfection, debridement, and adequate moisture to facilitate the natural healing process [11,12]. The pharmaceutical importance of plants lies in their bioactive compounds, which can enhance the healing and restoration of tissues by various mechanisms such as reducing oxidative stress, maintaining blood glucose levels, and collagen deposition [13]. These bioactive compounds belong to different chemical families, i.e., flavonoids, alkaloids, tannins, saponins, terpenoids, essential oils, and phenolic compounds [14]. Polyphenols have gained therapeutic importance mainly to promote wound healing. Generally, polyphenols possess strong antioxidant potential to protect against reactive oxygen species by neutralizing free radicals. Moreover, some polyphenols have antimicrobial potential against bacteria colonizing chronic wounds [15,16]. The presence of polyphenols in medicinal plants with high levels of antimicrobial, anti-inflammation, and antioxidant activities has encouraged scientists to explore their potential wound-healing effects [15].

*Moringa oleifera*, also called the miracle tree, is a widely cultivated species within the Moringaceae family. It is distributed all over the world, especially in Asia and Africa. Leaves, roots, flowers, and fruits of *M. oleifera* are edible and can be used as dietary supplements [17]. *Moringa oleifera* has been traditionally used for its anti-inflammatory [18], antioxidant [19], antifungal, antibacterial [20,21], antidiabetic [22], and healing activities [23]. This plant has also been used to treat hypertension, hypo-immunity, anemia, and other diseases [24]. *Moringa oleifera* leaf extract has been reported to promote the healing of infected wounds [25]. Studies have confirmed the presence of bioactive compounds in *M. oleifera* with beneficial health effects [26,27]. The leaves, however, are the most commonly used plant part which contains calcium, potassium, iron, proteins, vitamins E, C, and A, polyphenols, carotenoids, β-carotene, oxidase, alkaloids, isothiocyanates, tannins, and saponins [17,28,29]. *Moringa oleifera* dried leaves are rich in polyphenols, of which phenolic acids and flavonoids are most abundantly found [30]. Flavonoids and phenolic acids effectively scavenge oxygen free radicals and have antitumor effects [31,32]. The extraction of bioactive compounds from raw plant material is significantly affected by the drying temperature and type of the solvents used. However, the optimization of the extraction protocol, drying temperature, and choice of solvent is essentially required to improve the concentration of known compounds in plant extracts and also to maintain their biological activities [33]. 

The research is being conducted to evaluate the wound-healing effects of *M. oleifera* leaves dried at different temperatures, i.e., 10 °C, 30 °C, 50 °C, and 100 °C, and extracted in acetone, ethanol, and methanol. The extracts were analyzed using **a** DPPH (2,2-diphenylpicrylhydrazyl) assay, α-glucosidase inhibition assay, scratch assay, and MTT (3-(4, 5-dimethylthiazolyl-2)-2,5-diphenyltetrazolium bromide) assay. Moreover, the identification of major polyphenols present in the dried leaf extracts of *M. oleifera* was also performed using HPLC (high-performance liquid chromatography) analysis.

## 2. Results

### 2.1. α-Glucosidase Inhibition Assay

**Figure 1:**
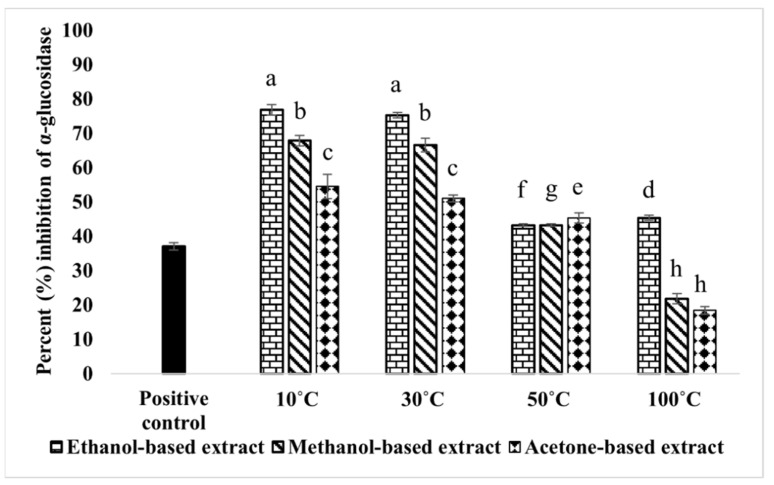
Effects of *M. oleifera* leaf extracts on α-glucosidase inhibition. The positive control was acarbose (10 mM). Bars displayed the mean based on 3 replicates of each treatment. Significant variations (*p* < 0.05) among all extracts are represented by different letters (a–h) above the bars.

The results of the inhibition of α-glucosidase by *M. oleifera* extracts confirmed that ethanol and methanol-based extracts showed the highest inhibition of α-glucosidase with 10 °C dried leaves, i.e., 76.82% and 75.23%, respectively. Acetone-based extracts displayed a moderate amount of activity as compared to other solvents. Likewise, 30 and 100 °C dried leaves followed the same order showing maximum inhibition with ethanol and minimum inhibition with acetone-based extracts. For extracts prepared with 50 °C dried leaves, there was no substantial difference in the percent inhibition of α-glucosidase among all the solvents (Figure 1). Overall, the highest activity was displayed by 10 °C dried leaves extracted in ethanol among all the tested extracts (IC_50_ value 0.05 mg/mL). The dose–response curves and IC_50_ values of the extracts showing the percentage inhibition above 50% at 0.1 mg/mL are represented in Figure S1. 

### 2.2. DPPH Assay

**Figure 2:**
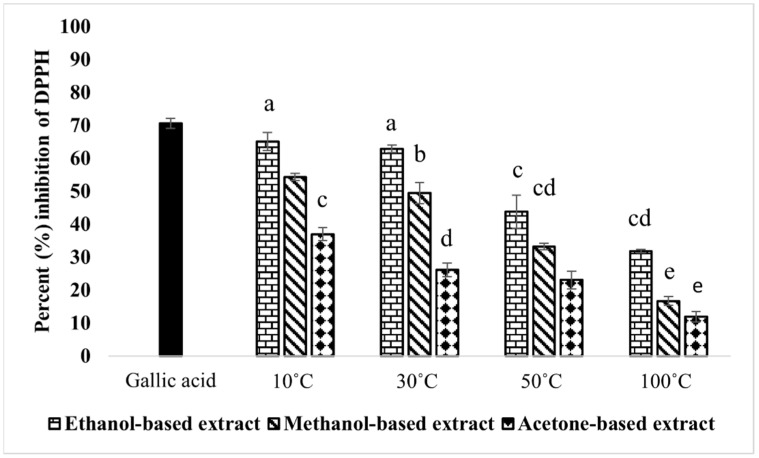
Antioxidant potential of *M. oleifera* leaves using DPPH assay. The positive control was gallic acid (0.3 mM). Bars displayed the mean based on 3 replicates of each treatment. Significant variations (*p* < 0.05) among all extracts are represented by different letters (a–e) above the bars.

*Moringa oleifera* extracts prepared by drying the leaves at variable temperature ranges and extracting in three solvents were tested for antioxidant activity by a DPPH assay (Figure 2). The results showed that the extracts prepared with 10 and 30 °C dried leaves extracted in methanol and ethanol had shown strong inhibitory potential against DPPH (greater than 50%) for the temperature studied. The leaves dried at 50 and 100 °C caused a significant reduction in the scavenging activity against DPPH compared to the gallic acid used as the positive control. For the solvents under study, substantial differences (*p* < 0.05) were seen among the different solvents, and ethanol-based extracts obtained the highest activity. The dose–response curves and IC_50_ of the extracts with percentage inhibitory activity above 50% at 0.1 mg/mL are shown in Figure S2.

### 2.3. MTT Assay

**Figure 3:**
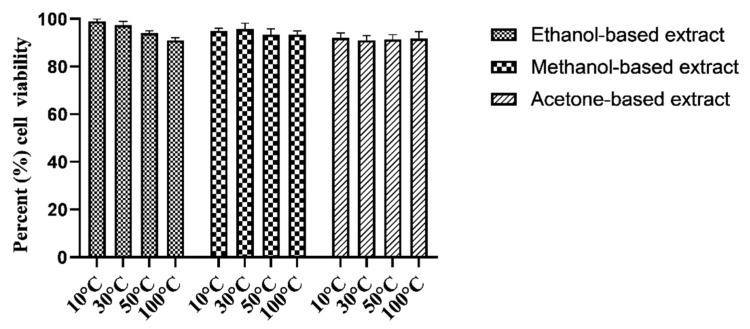
Retinal pigment epithelial cell viability after exposure to twelve different *M. oleifera* extracts prepared by drying the leaves at variable temperatures and extracting them in three different solvents. Extracts were tested at 0.1 mg/mL. Data are expressed as percent cell viability after treatment with plant extract. Bars displayed the mean ± standard deviation.

**Figure 4:**
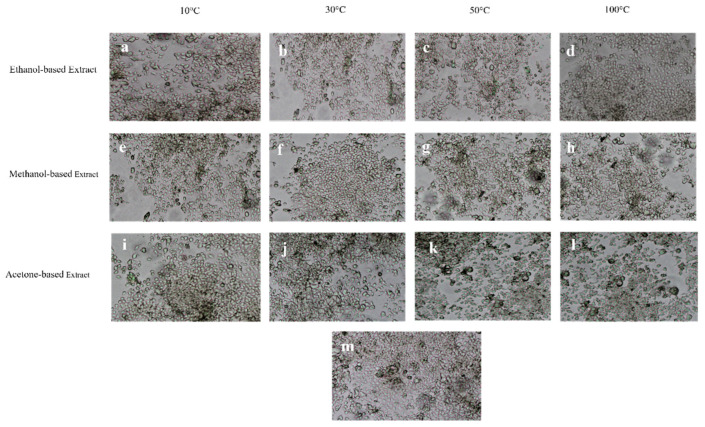
Cytotoxicity of 12 different *M. oleifera* extracts on Huh-7 cells after 48 h of exposure. (**a**–**d**) represent ethanol-based extract-treated cells, (**e**–**h**) are the methanol-based extract-treated cells, and (**i**–**l**) are acetone-based extract-treated cells. (**m**) represents PBS (used as negative control)-treated cells.

**Figure 5:**
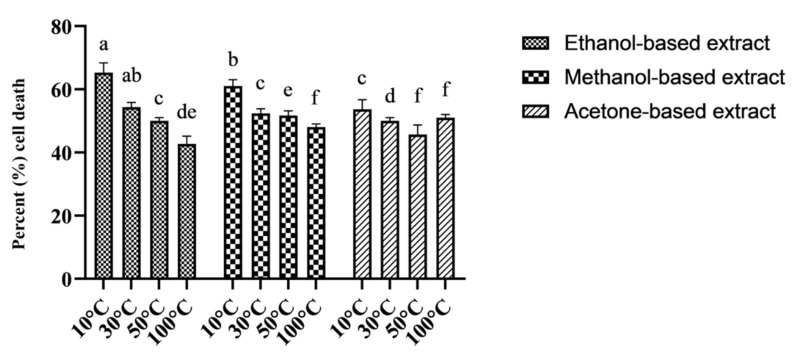
The cytotoxic effects of *M. oleifera* leaf extract on Huh-7 cells. Twelve different *M. oleifera* extracts were prepared by drying the leaves at variable temperatures and extracting them in three different solvents. Extracts were tested at 0.1 mg/mL. The data are expressed as the percent cell death of Huh-7 cells after treatment with plant extracts. Bars displayed the mean ± standard deviation. Significant variations (*p* < 0.05) among all extracts are represented by different letters (a–f) above the bars.

The evaluation of cell viability of RPE and Huh-7 cells after exposure to methanol, acetone, and ethanol-based *M. oleifera* extracts was performed using an MTT assay. All the extracts exhibited no toxicity on the RPE cells, as the percent viability of the cells remained at 91 to 99% (Figure 3). At the same time, the same extracts were cytotoxic for Huh-7 cells and caused a significant reduction in cell viability (Figure 4). Among the samples dried at variable temperatures, the extracts prepared by drying the leaves at 10 °C and 30 °C and extracting them in ethanol were more effective at inhibiting the cancer cell line growth (Figure 5).

### 2.4. Scratch Assay

**Figure 6:**
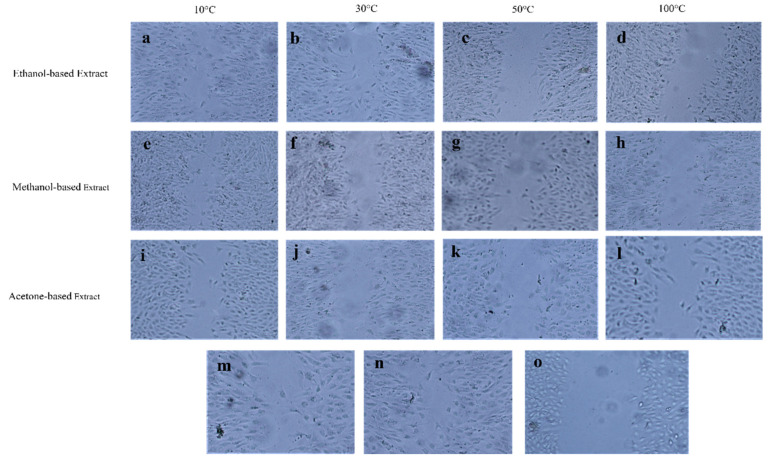
Wound healing activity of 12 different *M. oleifera* extracts at 16 h of incubation after the wound creation. (**a**–**d**) represent ethanol-based extract-treated cells, (**e**–**h**) are methanol-based extract-treated cells, and (**i**–**l**) are acetone-based extract-treated cells. (**m**–**n**) represents PBS and PDGF (used as negative and positive controls)-treated cells. (**o**) represents the scratch width at 0 h.

**Figure 7:**
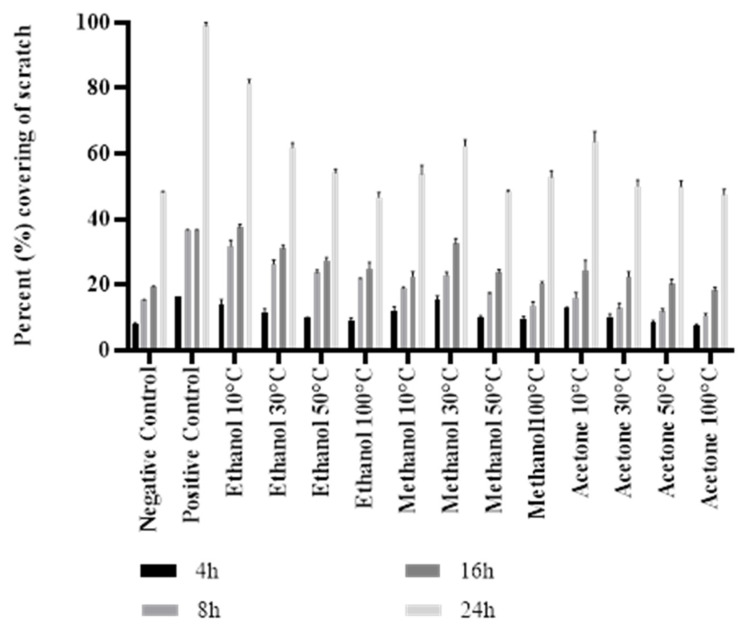
Cell proliferation activity of *M. oleifera* extracts on RPE cells. Twelve different *M. oleifera* extracts were prepared by drying the leaves at variable temperatures and extracting them in three different solvents at 0.1 mg/mL. Evaluations were conducted after different intervals, i.e., 4, 8, 16, and 24 h of incubation. The negative and positive controls were PBS and PDGF, respectively. Bars represent the mean ± standard deviation.

The healing potential of *M. oleifera* leaves on RPE cells using a scratch assay was investigated (Figure 6). After 24 h of exposure to each extract at 0.1 mg/mL concentration, it was seen that cell migration towards the artificially created scratch was induced. The analysis of the pictures captured at different time intervals has been represented as a graph (Figure 7, Table S1). The platelet-derived growth factor (PDGF as a positive control) showed the maximum wound closure rate of 99% after 24 h of treatment. The negative control exhibited the standard rate of healing without the effect of any treatment. Among the twelve different extracts, the 10 °C dried leaves extracted in ethanol-induced wound closure to a greater extent (81%) than the controls. An increase in the drying temperature significantly reduced the cell migration activity of *M. oleifera* leaves. The choice of solvent also considerably affected (*p* < 0.05) the healing of the scratch, and the activity was decreased in the order of ethanol > methanol > acetone-based extracts. 

### 2.5. HPLC Analysis

Polyphenol identification in ethanol-based *M. oleifera* leaf extracts was performed using HPLC analysis (Figure S3). The peaks were confirmed by the comparison of retention times with standards. Table 1 presents the identified compounds with their corresponding retention times. p-Coumaric acid, caffeic acid, and chlorogenic acid were found in very high concentrations in the extracts at 3.075, 7.494, and 2.88 min, respectively. At a lower temperature of 10 °C the amount of p-Coumaric acid was 346.49 mg/kg, chlorogenic acid was 228.43 mg/kg, and caffeic acid was 261.14 mg/kg. The concentrations of phenolic compounds identified in the tested extracts indicate significant differences among the extracts, with the highest amounts found at 10 °C. However, a decline in the number of polyphenols was seen with the temperature rise. The content of phenolic compounds potentially declines as the temperature rises to 100 °C. These results confirmed that the concentration of identified polyphenols and the biological activities determined in *M. oleifera* extracts are directly correlated.

## 3. Discussion

Wounds are a major healthcare concern, and the available therapeutic approaches cannot completely address the associated risk factors. There is an emerging demand to explore natural, biodegradable agents for wound healing as an alternative to conventional therapies [34]. Medicinal plants are being used to heal wounds because of their bioactive constituents, e.g., phenols, flavonoids, alkaloids, and triterpenes. These bioactive components of the plants have antimicrobial, anti-inflammation, and antioxidant properties and help to induce the collagen deposition and cell proliferation of keratinocytes and fibroblasts [2,35]. Recently, several reports on the potential effectiveness of polyphenols in the prevention and treatment of skin disorders, especially wound healing, have been published [36,37]. The evaluation of diverse polyphenol-rich plant extracts results in the development of innovative and cost-efficient wound-healing medications [38]. In this regard, *M. oleifera* leaves extracts were tested for antioxidant, antihyperglycemic, and cell migration properties assuming that this plant could promote wound healing in diabetic conditions.

Hyperglycemia is a major inhibiting factor of wound healing and is always accompanied by an increased level of inflammation and an imbalanced state of free radical production and antioxidant availability [39]. High blood glucose reduces cell migration [40] and collagen deposition [41] in various cells. The inhibition of α-glucosidase by phytochemical enriched plant extracts is an efficient strategy to cure hyperglycemia which can ultimately promote the healing of wounds in diabetic patients [42]. Extracts of *M. oleifera* were tested in the present study, suggesting that this plant could inhibit the α-glucosidase. The highest activity was obtained when leaves were dried at 10 and 30 °C. Increasing the temperature from 50 to 100 °C resulted in decreased activity. Moreover, ethanol-based extracts were more efficient than methanol- or acetone-based extracts, suggesting that both solvent and drying temperature may cause the changes in the chemical characteristics of the different extracts.

Oxidative stress is another risk factor associated with delayed wound healing. The excessive production of reactive oxygen species harms proteins, lipids, and DNA in cells, which promotes cellular and tissue dysfunction [43]. Plant-based antioxidants have been proven to have potent radical scavenging effects, thereby reducing the damage caused by free radicals during wound healing. Previous reports have supported the presence of polyphenols, i.e., gallic acid, quercetin, and kaempferol, with antioxidant potential in *M. oleifera* [44,45]. Our results suggested that *M. oleifera* leaves possess strong antioxidant potential. However, extracts prepared by drying the leaves at less than 50 °C, using ethanol and methanol as solvents, effectively scavenged the free radicals. 

Cell migration is one of the most essential phases of wound healing. The efficacy of numerous plant extracts on cell migration has been previously studied using a scratch assay [46,47]. In line with that, the effectiveness of *M. oleifera* leaf extracts on the cell migration of the RPE cell line was evaluated in this research. The study suggested that ethanolic *M. oleifera* extracts were highly effective in inducing cell migration towards the artificially created scratch. Plant extracts were also examined for cytotoxic effects through an MTT assay. The cytotoxic evaluation may help to determine the biological and therapeutic relevance of plant material [33]. In our study, *M. oleifera* extracts were non-toxic for RPE cells. After 48 h of exposure to leaf extracts, RPE cells still had a 91–99% viability rate, whereas the cancerous Huh-7 cells were highly affected after the extracts were applied. These findings demonstrated that *M. oleifera* extracts could be used as a wound-healing agent for further research.

Plant ethnomedicinal activities have been ascribed to polyphenolic and other bioactive compounds. The drying temperature and solvent used for extraction directly influence the polyphenol content and the biological activities of the extracts [48]. The recent literature has reported the effects of drying temperature on the antioxidant potential of thyme extracts. The study suggested that an increase in drying temperature causes a significant reduction in the antioxidant activity and total phenol content of the extracts [49]. Drying *Eucalyptus alba* leaves at high temperatures >50 °C causes the degradation of phenolic compounds and the reduced healing property of plant extracts [35]. However, a contradictory study has reported that the total phenolic content in turmeric rhizome was highest when dried at 100 °C [50]. Different solvents of high polarities are used to extract polyphenols from plant materials with high accuracy [48]. In line with that, *M. oleifera* leaves were dried at different temperatures and extracted in three different solvents to optimize the extraction of polyphenols.

Previous research has reported the phytochemicals, namely, kaempferol and quercetin in *M. oleifera* leaf extracts [51]. Another study reported the ethanolic extract of *M. oleifera* contained p-Coumaric acid and quercetin [52]. Chlorogenic acid, catechin, caffeic acid, rutin, quercetin, and kaempferol were identified in the leaves of *M. oleifera* using HPLC analysis [53]. *M. oleifera* leaves dried at four different temperatures were analyzed using high-performance liquid chromatography in the present research. Chlorogenic acid, p-Coumaric acid, caffeic acid, vanillic acid, kaempferol, sinapic acid, salicylic acid, coumarin, quercetin, and rutin were identified in ethanolic extract of *M. oleifera.* The results showed that the lowest concentrations of identified polyphenols were obtained in extracts dried at 100 °C, whereas the concentrations were higher in 10 and 30 °C -dried leaves. The difference in the concentrations of polyphenols in the plant materials could be due to the heat sensitivity of the compounds. The research outcomes are consistent with earlier studies, where drying at low temperatures resulted in the highest polyphenolic contents of *Aronia melanocarpa* stem extract [54].

## 4. Materials and Methods

### 4.1. Plant Sample Collection

Leaves of *M. oleifera* were obtained from AARI (Ayub Agricultural Research Institute, Faisalabad, Pakistan). A voucher specimen (B&BMOL-20-a) was submitted to the departmental herbaria. The plant name was affirmed by https://www.theplantlist.org (accessed on 16 May 2020). *Moringa oleifera* leaves were collected, washed, and allowed to dry in Memmert, Schwabach, Germany, in a convection oven at variable temperature ranges, i.e., 10, 30, 50, and 100 °C until they gained constant weight. For further analysis, the samples were finely ground and stored at −20 °C.

### 4.2. Plant Extract Preparation

The samples were extracted at room temperature. Briefly, the plant leaves (1 g from each sample) were soaked in pure methanol, acetone, and ethanol (10 mL) overnight with continual agitation. The solutions were sieved through filter paper (Whatman No.1). The solvents were vaporized at room temperature, and the dried mass was mixed in phosphate-buffered saline (PBS) to form 0.1 mg/mL of the final concentration [55,56]. To calculate the IC_50_ values for the antidiabetic and antioxidant activities, the extracts were diluted to 0.075, 0.05, 0.025, and 0.0125 mg/mL.

### 4.3. DPPH Assay

The antioxidant effects of *M. oleifera* extract were assessed using a DPPH assay. A stock solution of DPPH (0.3 mM) was formed in ethanol. Plant extract or gallic acid as a positive control (10 μL) and 190 μL of DPPH were pipetted into each well of 96-well plates following incubation of a half-hour in the dark. Optical density (OD) was measured using a 96-well plate reader (ELx808IU Biotek USA) at 517 nm. The percent inhibition of DPPH was calculated using OD values as follows:

Percent inhibition of DPPH = (OD of negative control − OD of sample)/OD of negative control × 100. 

### 4.4. α-Glucosidase Inhibition Assay

The inhibition of α-glucosidase by tested plant extracts was evaluated using 5 mM p-nitrophenyl-β-*D*-glucopyranoside (PNPG) as a substrate. A mixture of 40 μL of α-glucosidase (0.5 U/mL) was incubated with 12.5 μL of extract at varying concentrations with an additional 120 μL of PBS for 5 min. After incubation, 40 μL of 5 mM PNPG was added to the reaction mixture. The plate was placed at 37°C for a half-hour. Acarbose (10 mM) was used as the positive control and PBS as the negative control. Optical density was taken at 405 nm using a 96-well plate reader (ELx808 BioTek USA). The percent inhibition of α-glucosidase was calculated using OD values as follows:

Percentage inhibition = (OD of negative control − OD of sample)/OD of negative control × 100.

### 4.5. MTT Assay

The cell viability of human retinal pigment epithelial (RPE) and hepatocellular carcinoma (Huh-7) cells was determined upon the treatment of *M. oleifera* extract using an MTT assay. The cells were cultured at 2 × 10^4^ cells/well. The plate was placed in a CO_2_ incubator for 24 h in the above-stated conditions. The culture medium was replaced with fresh DMEM (Dulbecco’s modified Eagle medium) with 10 µL of tested plant extracts. The cells were again incubated for 48 h. After incubation, 5 mg/mL MTT solution was pipetted into each well, which was reduced to purple-color crystals of formazan by the viable cells. Dimethyl sulfoxide (DMSO) 150 µL was added to each well. The optical density was observed at 560 nm.

Percent cell death = (OD of negative control − OD of sample)/OD of negative control × 100.

Percent cell viability = 100 − Percent cell death

### 4.6. Scratch Assay

The migration rate of RPE cells after *M. oleifera* treatment was assessed using the scratch assay. Cells were cultured in DMEM containing penicillin/streptomycin (1%) and fetal bovine serum (10%) at a density of 2 × 10^5^ cells/well. The plate was incubated at favorable conditions, i.e., 5% CO_2_ and 37 °C, in a CO_2_ incubator. After 24 h of incubation, a monolayer of cells was scrapped using a sterilized 200 µL pipette tip to produce a linear wound. To remove the debris, the cells were rinsed with PBS. Fresh culture media with *M. oleifera* extracts was added and incubated for 24 h. Pictures were taken at 0, 4, 8, 16, and 24 h after the scratch creation using a Meiji Techno TC5200 inverted microscope at 40× magnification. Image analysis was performed with Image J 1.440 software for Windows at 1712 × 1368 pixels. The percent coverage of scratch was estimated as follows:

Percent wound healing = (Scratch width at nh − Scratch width at 0h)/Scratch width at nh × 100

where nh is the specific time interval at which calculations are performed.

### 4.7. HPLC Analysis

A Chromera HPLC system (PerkinElmer, Shelton, CT, USA) was used to analyze the polyphenol content in ethanol-based *M. oleifera* extracts. The system contained the Flexer Binary Liquid chromatography pump and a detector (UV/Vis), controlled by V.4.2.6410 software. The C-18 column containing an internal diameter of 250 × 4.6 mm and a particle size of 5 m was used to isolate polyphenols at 30 °C. The compounds were separated at a flow rate of 0.8 mL/min. The mixture of solvent A (30% methanol and 70% acetonitrile) and solvent B (0.5% glacial acetic acid and ddH_2_O) was used as the mobile phase. Peaks were identified by the comparison of retention time and the spike rate of the standards and samples. The external standard quantification method was used to quantify the compounds at 275 nm. The separation efficacy of HPLC was estimated by separation factor and resolution.

### 4.8. Calibration

For the purpose of calibration, standard solutions of pure compounds were prepared in the mobile phase at 100, 200, 400, 600, 800, and 1000 µg/mL. Graphs were created by drawing the peak area of each compound against the concentration.

### 4.9. Statistical Analysis

The experiments were conducted in triplicate, and the data were presented as mean ± standard deviation. Significant comparisons (*p* < 0.05) among different groups were created by two-way ANOVA and Tukey’s post hoc test using GraphPad prism.

## 5. Conclusions

The effects of variable drying temperatures and extraction solvents on the polyphenolic composition and diabetic wound-healing activity of *Moringa oleifera* were observed in the present research. The outcomes suggested that extraction solvent and drying temperature significantly affected (*p* < 0.05) the cell migration, α-glucosidase inhibition, and antioxidant activities of *M. oleifera* extracts. Further, the plant extract showed no signs of toxicity on retinal pigment epithelial cells, and the same extracts were cytotoxic for hepatocellular carcinoma cells. Phenolic compounds identified in the tested extracts indicate significant differences among the extracts, with the highest amounts being found at 10 °C and the lowest amounts at 100 °C. These results confirmed a direct relationship between the concentration of identified polyphenols and the biological activities of *M. oleifera* extracts. Based on the findings of this study, *M. oleifera* leaf extracts may have the potential for diabetic wound healing when extracted at optimum conditions. However, future research is needed to determine the specified compounds involved in the wound-healing efficacy of *M. oleifera.*

## Figures and Tables

**Table 1:** Polyphenols identified in ethanol-based *M. oleifera* leaf extracts.

Polyphenols	Retention Time (min)	Concentration (mg/kg) in *M. oleifera* 10 °C Dried Leaves	Concentration (mg/kg) in *M. oleifera* 30 °C Dried Leaves	Concentration (mg/kg) in *M. oleifera* 50 °C Dried Leaves	Concentration (mg/kg) in *M. oleifera* 100 °C Dried Leaves
**Chlorogenic acid**	2.880	228.43	225.01	130.93	120.54
**p-Coumaric acid**	3.075	346.49	288.82	267.02	14.03
**Caffeic acid**	7.494	261.14	203.74	198.83	8.38
**Vanillic acid**	7.687	19.45	7.47	7.56	18.55
**Kaempferol**	11.074	20.17	4.08	2.01	ND
**Sinapic acid**	12.237	36.83	34.74	26.02	ND
**Salicylic acid**	15.296	34.82	33.42	ND	2.36
**Coumarin**	16.085	69.02	65.15	ND	ND
**Quercetin**	16.954	63.19	45.34	ND	ND
**Rutin**	23.989	54.23	32.19	3.13	ND

## Data Availability

Not applicable.

## Supplementary Materials

The following supporting information can be downloaded at: https://www.mdpi.com/article/10.3390/molecules28020710/s1.

## Sample Availability

Samples of the compounds are available from the authors.
