# Supplementary material for: Effects of Drying Temperature and Solvents on In Vitro Diabetic Wound Healing Potential of Moringa oleifera Leaf Extracts
Source: Molecules. 2023 Jan 11;28(2):710. doi: 10.3390/molecules28020710 (PMC9864430; doi:10.3390/molecules28020710)
Supplement: Supplementary file 1 [file molecules-28-00710-s001.zip › molecules-2082775-supplementary.pdf]

Table S1. Cell proliferation activity of *M. oleifera* extracts on RPE cells. Evaluations were conducted after different intervals i.e., 4, 8 16 and 24 hours of incubation. Negative control and positive control were PBS and PDGF respectively.

	**4 hours**	**8 hours**	**16 hours**	**24 hours**
Negative Control	8.29 c	15.29 g	19.48 f	48.29 f
Positive Control	16.62 a	36.80 a	36.81 a	99 a
Ethanol 10°C	14.04 b	31.78 b	37.51 a	81.52 b
Ethanol 30°C	11.65 b	26.40 c	31.15 c	61.87 c
Ethanol 50°C	9.92 c	23.60 c	27.42 d	54.20 d
Ethanol 100°C	9.01 c	21.87 cd	24.96 e	46.66 f
Methanol 10°C	11.99 b	18.98 e	22.53 ef	53.80 d
Methanol 30°C	15.48 a	22.86 c	32.64 b	62.35 c
Methanol 50°C	10.01 c	17.30 f	23.78 e	48.35 f
Methanol100°C	9.43 c	13.66 g	20.51 f	52.85 d
Acetone 10°C	12.94 b	16.06 fg	24.41 e	63.74 c
Acetone 30°C	10.02 c	12.77 g	22.44 ef	50.11 e
Acetone 50°C	8.33 c	11.84 gh	20.21 f	49.99 e
Acetone 100°C	7.47 c	10.64 h	18.3 g	47.53 f

a

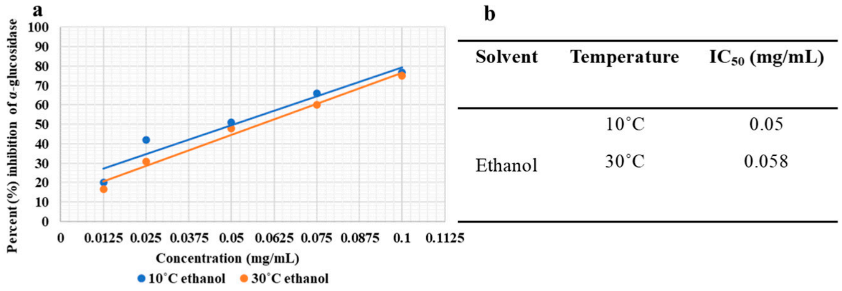

b

Solvent	Temperature	IC_{50} (mg/mL)
Ethanol	10˚C	0.05
	30˚C	0.058

c

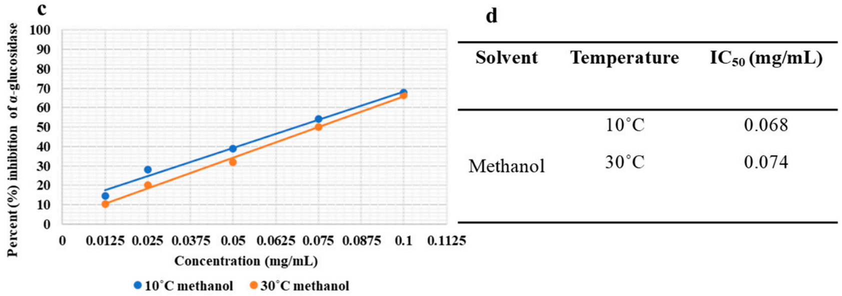

d

Solvent	Temperature	IC_{50} (mg/mL)
Methanol	10˚C	0.068
	30˚C	0.074

e

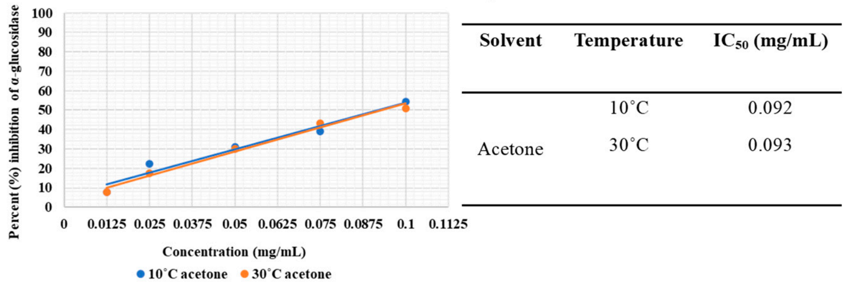

f

Solvent	Temperature	IC_{50} (mg/mL)
Acetone	10˚C	0.092
	30˚C	0.093

Figure S1. Dose-response and IC_{50} value of ethanol-based extract (a-b), methanol-based extract (c-d), and acetone-based extract (e-f) of *M. oleifera* leave.

a

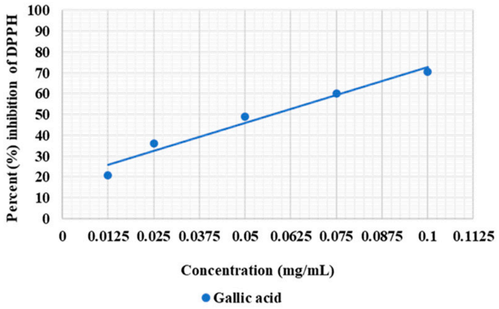

b

IC_{50} (mg/mL)
0.057

c

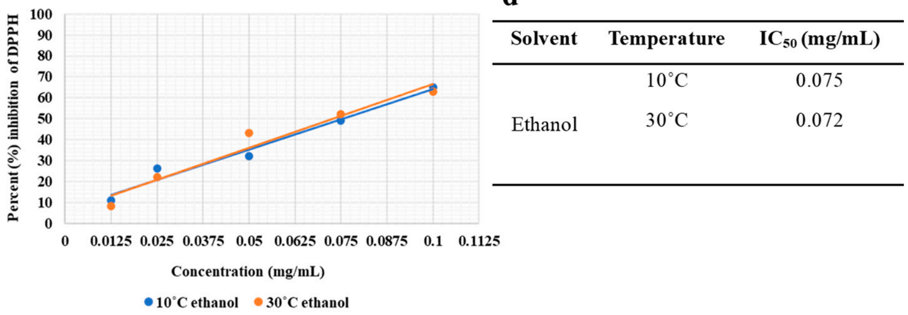

d

Solvent	Temperature	IC_{50} (mg/mL)
Ethanol	10˚C	0.075
	30˚C	0.072

e

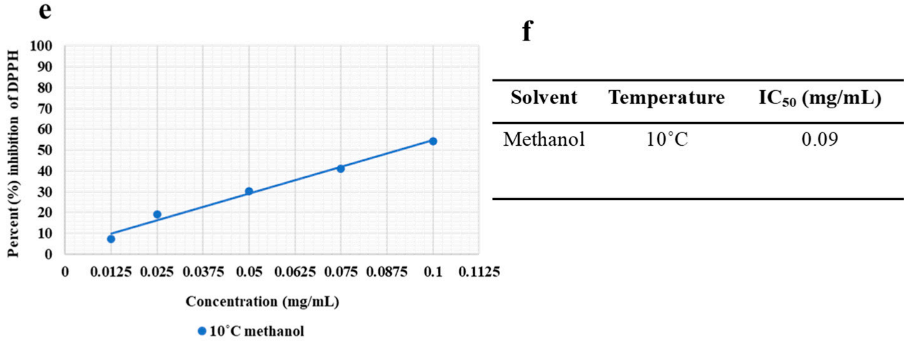

f

Solvent	Temperature	IC_{50} (mg/mL)
Methanol	10˚C	0.09

Figure S2. Dose-response and IC_{50} value of gallic acid (a-b) ethanol-based extract (c-d) and methanol-based extracts (e-f) of *M. oleifera* leaves.

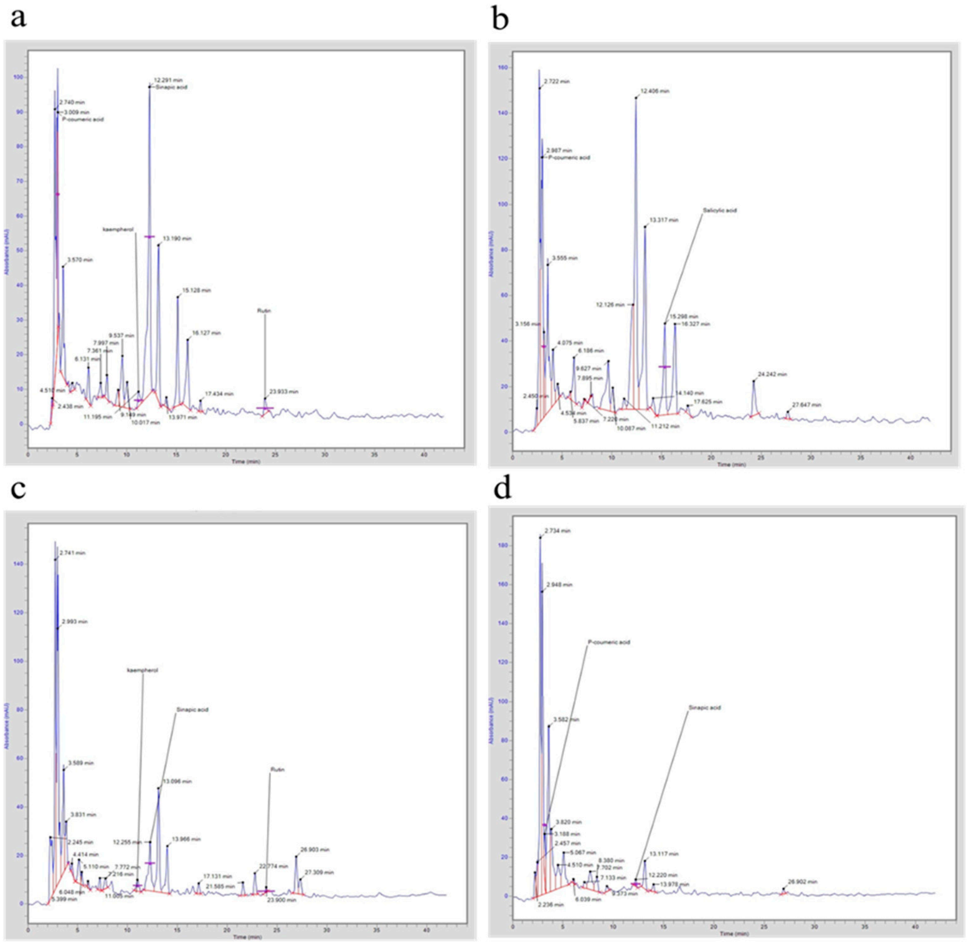

Figure S3. HPLC chromatogram of ethanol-based *M. oleifera* leaf extracts dried at **(a)** 10°C, **(b)** 30°C, **(c)** 50°C, **(d)** 100°C.
